# Enhanced GABAergic Inhibition of Cholinergic Interneurons in the zQ175^+/−^ Mouse Model of Huntington's Disease

**DOI:** 10.3389/fnsys.2020.626412

**Published:** 2021-01-20

**Authors:** Sean Austin O. Lim, D. James Surmeier

**Affiliations:** ^1^Department of Physiology, Feinberg School of Medicine, Northwestern University, Chicago, IL, United States; ^2^Neuroscience Program, College of Science and Health, DePaul University, Chicago, IL, United States

**Keywords:** channelrhodopsin (ChR2), giant aspiny interneurons, dorsal striatum, paired-pulse ratio, GABA uncaging, medium spiny neuron, striatal interneuron, pacemaker activity

## Abstract

Huntington's disease (HD) is an autosomal dominant neurodegenerative disorder that initially manifests itself in the striatum. How intrastriatal circuitry is altered by the disease is poorly understood. To help fill this gap, the circuitry linking spiny projection neurons (SPNs) to cholinergic interneurons (ChIs) was examined using electrophysiological and optogenetic approaches in *ex vivo* brain slices from wildtype mice and zQ175^+/−^ models of HD. These studies revealed a severalfold enhancement of GABAergic inhibition of ChIs mediated by collaterals of indirect pathway SPNs (iSPNs), but not direct pathway SPNs (dSPNs). This cell-specific alteration in synaptic transmission appeared in parallel with the emergence of motor symptoms in the zQ175^+/−^ model. The adaptation had a presynaptic locus, as it was accompanied by a reduction in paired-pulse ratio but not in the postsynaptic response to GABA. The alterations in striatal GABAergic signaling disrupted spontaneous ChI activity, potentially contributing to the network dysfunction underlying the hyperkinetic phase of HD.

## Introduction

Huntington's disease (HD) is an autosomal dominant neurodegenerative disorder caused by a CAG expansion in the Huntingtin gene (McColgan and Tabrizi, 2018). The disease is characterized by progressive cognitive disability and deterioration of motor control (Ross and Tabrizi, 2011). In adult onset HD, early in the course of the disease, hyperkinetic motor symptoms, like chorea, typically predominate. As the disease progresses, these manifestations of the disease are supplanted by hypokinetic deficits, like bradykinesia and dystonia.

The motor symptoms of HD are attributable in large measure to pathology in the striatum, a key structure of the basal ganglia (Waldvogel et al., 2015; Plotkin and Goldberg, 2018). The principal neurons of the striatum are GABAergic, spiny projection neurons (SPNs). SPNs can be divided into two classes based upon their axonal projections: indirect pathway SPNs (iSPNs) and direct pathway SPNs (dSPNs). The differences in axonal projection are strongly correlated with a broad array of other cellular characteristics, including the expression of G-protein coupled receptors (GPCRs), somatodendritic architecture and intrinsic excitability (Parent et al., 1984; Kreitzer and Malenka, 2007; Shen et al., 2008; Gerfen and Surmeier, 2011). In HD, iSPNs are particularly vulnerable and manifest signs of hypoexcitability early in the course of the human disease and early in the evolution of the phenotype in mouse models of HD, including the zQ175 knockin model of HD (Deng et al., 2004; Galvan et al., 2012; Goodliffe et al., 2018; Reiner and Deng, 2018). Given the role of iSPNs in suppressing unwanted movement (DeLong, 1990; Bateup et al., 2010; Kravitz et al., 2010), this sequencing aligns with the early dominance of hyperkinetic symptoms in HD.

Recently, it was demonstrated that the synaptic connectivity of iSPNs with neurons in the external segment of the globus pallidus (GPe) is enhanced in zQ175 mice (Perez-Rosello et al., 2019). The alteration in connectivity was progressive, appearing in parallel with motor symptoms in this model. In addition to innervating GPe neurons, iSPNs have collateral projections to other SPNs and striatal interneurons (Taverna et al., 2008; Gonzales et al., 2013; Lim et al., 2014). Among the striatal interneurons, cholinergic interneurons (ChIs) are prominent targets. ChIs are key modulators of the striatal circuitry underlying motor control, serving to enhance activity in iSPNs and suppress it in dSPNs. In so doing, they facilitate suppression of inappropriate movement and set-shifting (Bradfield et al., 2013; Aoki et al., 2015). In HD patients and in HD models, striatal acetylcholine release is impaired (Smith et al., 2006; Farrar et al., 2011). Precisely why this is the case is unclear, but one contributing factor could be enhanced GABAergic inhibition of ChIs by iSPN collaterals, much as seen at the striatopallidal synapse.

To test this hypothesis, the synaptic connectivity of iSPNs and dSPNs was examined using optogenetic and electrophysiological approaches in *ex vivo* brain slices from presymptomatic and symptomatic heterozygous zQ175^+/−^ mice. These studies revealed a several fold increase in the magnitude of GABAA receptor mediated currents generated by stimulation of iSPN collaterals in slices from symptomatic, but not presymptomatic, zQ175 mice. In contrast, collateral input arising from dSPNs was unchanged. The change in strength of the iSPN collateral input to ChIs was sufficient to induce a pronounced pause in their autonomous activity monitored in cell-attached recordings to preserve physiological Cl- gradients. The locus of the change was presynaptic, as the enhancement in GABAergic transmission was accompanied by an elevation of release probability. Together, these results suggest that iSPN hypoexcitability in the zQ175 HD model triggers synaptic adaptations that serve to compensate for the activity deficit. However, in so doing the adaptation may induce secondary deficits in the timing of ChI activity and striatal ACh release, leading to broader striatal dysfunction.

## Materials and Methods

### Animals

The following studies were conducted in accordance with the guidelines set forth by the Northwestern University Animal Care and Use Committee. To model Huntington's disease (HD), we used male mice that were heterozygous for the zQ175 knock-in (zQ175^+/−^) with an expanded polyglutamine sequence of ~190 repeats on a C57/Bl6 background (B6J.zQ175DN KI mice (Jackson Labs; Stock No. 029928). This experimental group was compared with zQ175 WT (–/–) mice that expressed no abnormal polyglutamine expansion.

Mice were weighed immediately before sacrifice. Six-9 month-old male HD mice were significantly lighter than WT mice. The median weight of a WT mouse was 33.71 g, while the median weight of an HD mouse was 31.34 g [Mann Whitney U = 705, n(WT) = 46 mice, n(HD) = 46 mice; *P* = 0.0055 two-tailed].

### Electrophysiology

Mice were anesthetized with isoflurane before receiving an i.p. injection of ketamine (100 mg/kg)/xylazine (7 mg/kg). Mice remained outside of the home cage until a noxious foot pinch failed to elicit a response, and were then transcardially perfused with ice cold solution consisting of, in mM: NaCl 125, KCl 2.5, NaHCO_3_ 25, NaH_2_PO_4_ 1.25, CaCl_2_ 1, MgCl_2_ 1.5, Glucose 20. Brains were rapidly extricated from the skull, the cerebellum separated and removed, then divided along the midline. Two hundred fifty micrometer thick parasagittal slices were cut using a vibratome (Leica VT 1200 S). Slices containing the lateral half of the striatum were used for recording. Slices were placed immediately into room temperature bubbled (95% O_2_, 5% CO_2_) ACSF containing in mM: NaCl 125, KCl 2.5, NaHCO_3_ 25, NaH_2_PO_4_ 1.25, CaCl_2_ 2, MgCl_2_ 1, Glucose 10. Slices were allowed to recover for at least 1 h before being transferred to a 34°C recording chamber, where they were superfused with bubbled ACSF (2–4 mL/min).

**Figure 1:**
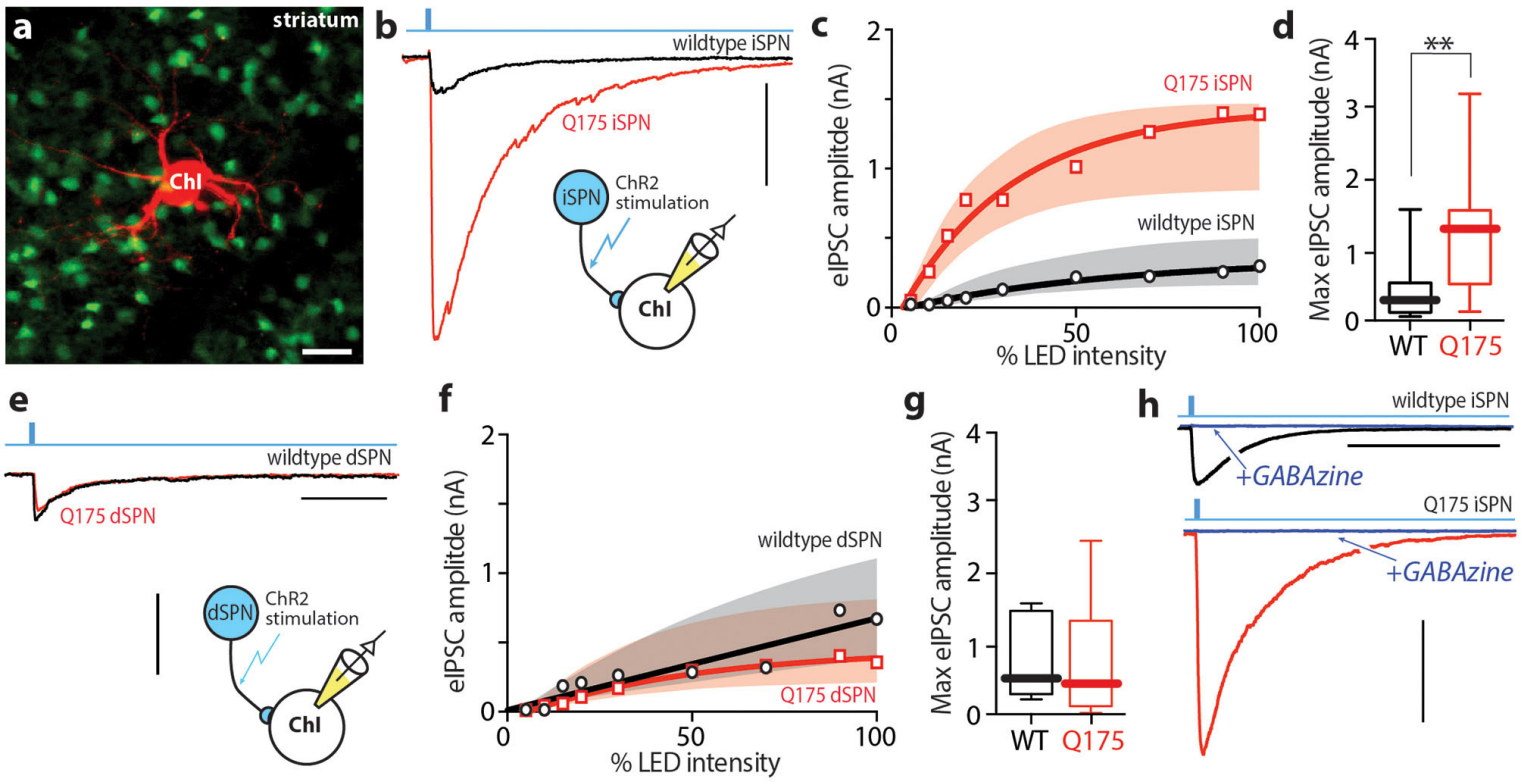
iSPN derived GABAergic signaling onto ChIs is enhanced in 6–9 month old zQ175^+/−^ HD mice. **(a)** Confocal image showing the giant cholinergic interneuron (red) and several iSPNs in the dorsolateral striatum. **(b)** Sample traces taken from WT (black) and zQ175^+/−^ (red) ChIs after inducing optogenetic release of GABA from iSPNs. Scale bar = 0.5 nA. **(c)** Summary of input - output relationship of eIPSC amplitude at the iSPN-ChI synapse at progressively higher optical stimulation intensities. **(d)** Box plots of peak eIPSC amplitude at the iSPN-ChI synapse at maximum stimulation intensity. **(e)** Sample traces taken from WT (black) and zQ175^+/−^ (red) ChIs after inducing optogenetic release of GABA from dSPNs. Scale bar = 0.5 nA; 100 ms. **(f)** Summary of input - output relationship at the dSPN-ChI synapse. **(g)** Box plots of peak eIPSC amplitude at the dSPN-ChI synapse at maximum stimulation intensity. **(h)** Addition of SR95531 hydrobromide (GABAzine; blue) completely blocks optogenetic response at the iSPN-ChI synapse in both WT and zQ175^+/−^ mice. Scale bar = 0.5 nA; 500 ms. **P < 0.01.

ChIs were visualized with an epifluoresence microscope under high power magnification (water-immersion 40x Olympus LUMPlan FI/IR). ChIs have large cell bodies around 25–30 μm in diameter (compared with other striatal cells of diameter <15 μm), are aspiny, and often have one or two thick processes branching off the soma (Figure 1a) (Bolam et al., 1984; Gonzales and Smith, 2015). Upon obtaining electrical access to the cell, electrophysiological characteristics were used to distinguish ChIs from other striatal cell populations (Cepeda et al., 2008; Arias-Garcia et al., 2018). Only ChIs located in the dorsal half of the striatum and rostral to the anterior commissure decussation were included for analysis.

Borosilicate glass electrodes of resistance 2–4 MOhm (Sutter Instrument, 1.5 mm O.D., 0.86 I.D., 10 cm length) were made with a horizontal puller (Sutter Instrument, Model P-1000 Flaming/Brown micropipette puller). For voltage clamp recordings, the internal solution contained in mM: CsCl 120, NaCl 10, HEPES 10, QX314 Br 2, ATP Mg 4, GTP Na 0.3, EGTA 0.2. Cells were held at −60 mV in voltage clamp, and following rupture of the cell membrane, were allowed to equilibrate for at least 5 min before recording. For cell attached measures in current clamp, the pipettes were filled with filtered ACSF. In cell attached recordings, a loose patch of resistance 50 MOhm or less was preferred. For the measurement of asynchronous evoked inhibitory postsynaptic currents (IPSCs), the external solution was substituted with calcium-free strontium ACSF +DNQX +APV containing the following: NaCl 125, KCl 2.5, NaHCO_3_ 25, NaH_2_PO_4_ 1.25, SrCl_2_ 3.2, MgCl_2_ 1, Glucose 10, DNQX 0.01, APV 0.05. Slices were incubated in this ACSF for at least 20 min before recordings were taken.

Recordings were obtained using a MultiClamp 700 A amplifier. Signals were digitized at 10 kHz and filtered at 2 kHz. Voltage clamp recordings with an access resistance >25 MOhm or a change in access resistance >20% were eliminated from analysis.

### Stereotaxic Delivery of Viral Vectors

*Adora2*-cre x zQ175, *Drd1*-cre x zQ175, NPY-cre x zQ175, or PV-cre zQ175 mice were anesthetized lightly with isoflurane (Model 100 vaporizer, SurgiVet) before being placed in a stereotaxic frame (Kopf model 962). The front teeth were placed in a holding apparatus, and ear bars were used to restrain the head. Metacam (2 mg/kg) in saline were delivered subcutaneously in the hind flank to decrease inflammation and maintain hydration, and ophthalmic ointment (Artificial tears, Henry Schein) was spread over the eyeballs to minimize drying. The surface area above the skull was sanitized with three alternating alcohol wipes and an iodine swabstick. A 2–3 cm caudal to rostral incision was made above the skull along the midline, and the skin was pulled laterally to expose the surface of the skull. The injection site in dorsolateral striatum (−2.2 mediolateral, 0.5 anteroposterior, between −2.9 and −3.2 dorsoventral) was targeted using a computer-aided stereotaxic system (Leica, Angle Two). After drilling a burr hole (L12M driven by EXL M40, Osada) in the skull above the target injection site, a virus-filled pipette was lowered (1 cm/min) until the dorsoventral coordinate was reached. Virus was injected at a uniform speed (Narashige IM 300 microinjector), and to minimize the uncontrolled spread of the virus, the pipette was held at the coordinates for 5 min before slow (1 cm/min) withdrawal. The incision site was stitched using a monofilament polypropylene suture (5–0, Henry Schein), and removed after 2 weeks. Lidocaine (2.5%)/prilocaine (2.5%) cream was spread liberally over the surgery site, and the mouse was allowed to recover on a heated pad for an hour before being returned back to the home cage. Anesthesia level was monitored throughout surgery.

A cre-dependent AAV9-EF1a-DIO-hChR2(H134R)-eYFP (Addgene 20298, 2.11 × 1,013 gc/ml) construct was used to induce expression channelrhodopsin among cre-positive cells, and a Chronos channelrhodopsin AAV9-hSyn-FLEX-Chronos-GFP (Addgene 62722, 6.5 × 10^∧^12) was used for obtaining paired-pulse ratio data. Viral constructs were diluted 1:5 in saline before injecting a total volume of 1 μL into dorsolateral striatum. AAV containing ChR2 was allowed to express for 2–4 weeks before recording, whereas AAV containing Chronos was given 6–7 weeks.

For mice labeled “Adora2A-cre x Q175,” a combination of the viral injected Adora2A-cre × Q175 mice and the triple cross Ai32 × Adora2A-cre × Q175 mice with cre-restricted expression of a channelrhodopsin-2/EYFP fusion protein were used unless otherwise stated. The findings were reproduced in both genotypes (Supplementary Figures 1C,D), thus the data were combined.

### Optogenetics

Four hundred and seventy nanometer blue light pulses were delivered *via* full field illumination (CoolLED pE-100). Pulse duration was 0.5 ms, and stimulation was done under 40x power magnification. In cell attached current clamp recordings, a high frequency train (maximum light output intensity, 10 pulses, 0.5 ms in duration at 50 Hz) stimulation was applied.

To collect input-output data, peak evoked IPSC amplitude was measured in response to a single LED flash at progressively higher intensities (5, 10, 15, 20, 30, 50, 70, 90, 100%, corresponding to ~2.5, 5, 8.5, 12, 15, 23, 33, 43, and 46 mW, respectively). For PPR analysis and asynchronous release experiments, stimulation intensity was titrated so eIPSC amplitude was between 100 and 300 pA, or half maximum eIPSC amplitude, whichever was smaller. Cells with unclamped spiking currents that affected peak amplitude, or traces with spontaneous events that overlapped with the onset of the evoked response were excluded from analysis.

### Cell Morphology and Confocal Imaging

Biocytin (1 mg/mL) was added into the internal solution. Minimal positive pressure was applied while searching for cells to minimize dispersal of biocytin. Visually identified ChIs were patched, and after 40 min, the patch pipette was pulled away from the cell rapidly. An additional 15 min elapsed to allow the cell membrane to repair itself before slices were then transferred into 4% paraformaldehyde for at least 1 h. Up to five rinses with PBS were performed before incubating the slice overnight with secondary antibody Alexa 568 in a blocking and permeabilizing solution containing Triton X, normal goat serum, bovine serum albumin, and tris-buffered saline. Slices were rinsed with PBS 5 times, mounted onto glass slides in the same orientation as during filling, and imaged using a confocal microscope.

### Pharmacology

6,7-dinitroquinoxaline-2,3-dione (DNQX) 10 μM, (2R)-amino-5-phosphonovaleric acid (APV) 50 μM (HelloBio), SR95531 hydrobromide (Gabazine) 10 μM (Tocris), Quinpirole 1 μM (Tocris), and RuBiGABA 10 μM (Tocris) were bath applied for at least 5 min before recording. For end of recording verification of GABA transmission, a saturating concentration of SR95531 (10 μM) was added directly into the recording chamber.

### Data Analysis and Statistics

No assumptions were made about normality of distribution, so nonparametric statistics tests were performed. For input/output graphs, the thick line represents the median and the shaded areas represent the upper and lower quartiles as fitted by a single exponential curve. For maximum eIPSC amplitude graphs, boxplots are used to represent data, where the thick bar represents the median, the box edges are the upper and lower quartiles, and the whiskers are the range of data points. Miniature IPSCs were analyzed using MiniAnalysis 6.0 (Synaptosoft), statistical analysis was carried out using Prism 6 (GraphPad Prism version 6.0 for Mac OS X, GraphPad Software, La Jolla California USA, www.graphpad.com), and plots were generated with a combination of Prism 6 and Illustrator (Adobe Systems).

## Results

### iSPN but Not dSPN Collateral GABAergic Signaling to ChIs Was Enhanced in HD Mice

To assess the strength of SPN-mediated inhibition of ChIs in heterozygous zQ175 (zQ175^+/−^) mice, a combination of optogenetic and electrophysiological methods was used. To achieve selective expression of channelrhodopsin 2 (ChR2) in iSPNs or dSPNs, zQ175^+/−^ mice were crossed with Adora2-Cre or Drd1-Cre mice, allowing Cre recombinase (Cre) dependent gene expression in iSPNs or dSPNs, respectively (Fuxe et al., 2007; Lobo and Nestler, 2011). In zQ175^+/−^ het mice and wildtype littermate controls, an adeno-associated virus (AAV) was stereotaxically injected into the dorsolateral striatum and 2–4 weeks later mice were sacrificed and *ex vivo* brain slices prepared for study using standard methodologies. Giant, aspiny ChIs were visually identified and subjected to whole cell patch clamp recording using a Cs^+^-based internal solution to maximize voltage clamp (Figure 1a). Ionotropic glutamate receptors (α-amino-3-hydroxy-5-methyl-4-isoxazolepropionic acid receptors - AMPARs; N-methyl-d-aspartate receptors – NMDARs) were pharmacologically antagonized with bath application of 10 μM DNQX and 50 μM APV, and this inhibition did not change the amplitude of evoked IPSC (Supplementary Figures 1A–C). SPN collaterals were optogenetically activated with full field, 470 nm blue flash (0.5 ms) while voltage-clamping ChIs at −60 mV.

Optogenetic activation of iSPN collaterals in *ex vivo* brain slices from 6 to 7 month-old *Adora2*-cre X zQ175^+/−^ mice yielded significantly larger evoked inhibitory postsynaptic currents (eIPSCs) than in slices from age-matched, wildtype littermate controls (Figure 1b). This difference was seen across a range of optical stimulation intensities (Figure 1c). At saturating optical intensity, the median eIPSC amplitude in wildtype ChIs was 264 pA (*n* = 13 cells, 8 mice), whereas the median eIPSC amplitude in zQ175^+/−^ ChIs was nearly 5-fold larger (1,301 pA, *n* = 15 cells, 7 mice; Mann-Whitney U = 39, *P* < 0.0061 two-tailed; Figure 1d). To control for variation in AAV transduction, this result was replicated in mice crossed into the Ai32 line in which ChR2 expression is controlled by Cre expression (Madisen et al., 2012) (Supplementary Figures 1D,E). This observation was specific to iSPN collaterals; in contrast, the eIPSCs in Drd1-cre x wildtype and zQ175^+/−^ ChIs evoked by stimulation of dSPN collaterals were not significantly different at any stimulus intensity (Figures 1e,f). At saturating optical intensity, the median eIPSC amplitude in wildtype ChIs was 477 pA (*n* = 4 cells, 5 mice), while the median eIPSC amplitude in zQ175^+/−^ ChIs was 447 pA (*n* = 5 cells, 4 mice; Mann Whitney U = 9, *P* = 0.873 two-tailed; Figure 1g). In both wildtype and zQ175^+/−^ ChIs, the evoked responses were completely blocked with the GABA_A_ receptor (GABA_A_R) antagonist SR95531 (10 μM) (Figure 1h).

### The Change in GABAergic Signaling Paralleled the Expected HD Phenotype

**Figure 2:**
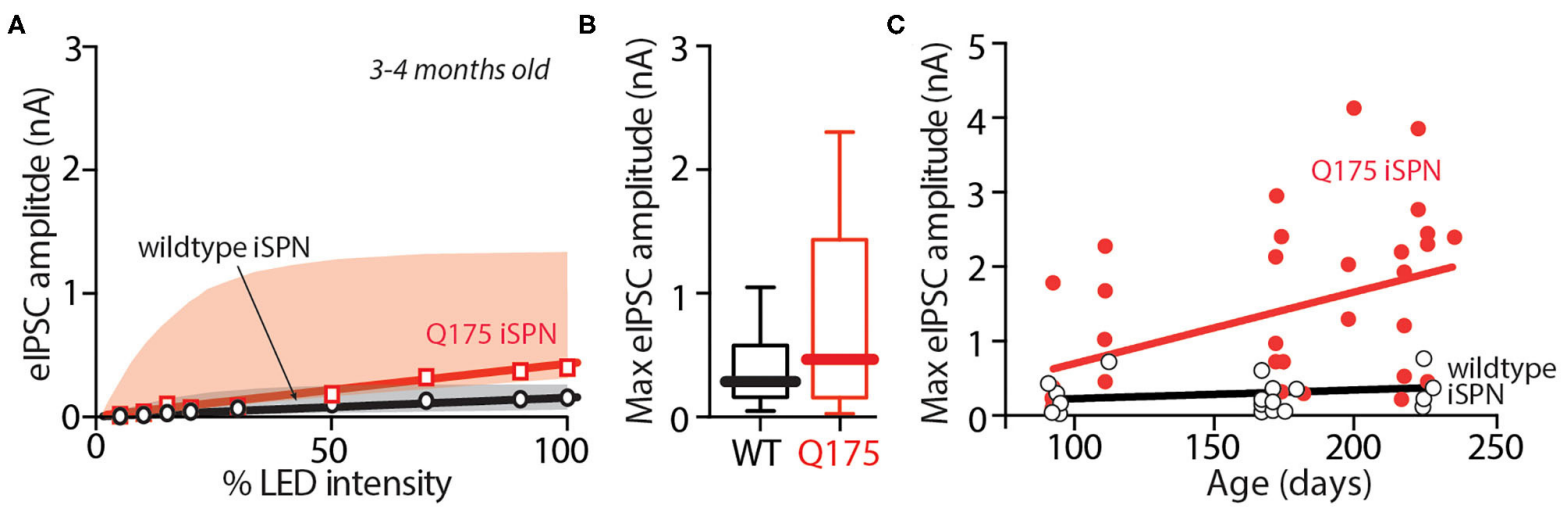
In zQ175^+/−^ mice, iSPN-ChI GABA signaling is enhanced in 6–9 month old mice, but not in 3–4 month old mice. **(A)** Summary of input - output relationship of eIPSC amplitude at the iSPN-ChI synapse at progressively higher stimulation intensities in young zQ175^+/−^ (red) and WT (black) mice. **(B)** Box plots of maximum eIPSC amplitude in WT and zQ175^+/−^ mice aged 3–4 months showing no significant statistical difference between genotypes. **(C)** Maximum eIPSC amplitude increases linearly with age in ChIs from zQ175^+/−^ mice, but not in ChIs from WT mice.

In zQ175^+/−^ mice, deficits in motor performance become clearly evident at about 6 months of age (Menalled et al., 2012; Smith et al., 2014). To determine whether there was a correlation between the emergence of motor deficits and the change in iSPN collateral GABAergic synaptic transmission, presymptomatic, 3–4 month-old zQ175^+/−^ mice were compared with symptomatic 6–9 month-old zQ175^+/−^ mice. In these experiments, crosses into the Ai32 ChR2 reporter line were used. iSPN collateral eIPSCs in young zQ175^+/−^ ChIs were not significantly different from those evoked in age-matched, wildtype ChIs across a range of stimulus intensities (Figure 2A). At maximum stimulation intensity, the median amplitude of the eIPSC in young ChIs was 281 pA in wildtype ChIs and 484 pA in zQ175^+/−^ ChIs [Mann Whitney U = 71, *n*(wildtype) = 15 cells, 5 mice, *n*(zQ175^+/−^) = 13 cells, 4 mice; *P* = 0.2332 two-tailed; Figure 2B]. To better visualize the relationship between eIPSC amplitude and age in the two groups, peak amplitudes were plotted against age for both young and older mice (Figure 2C). While linear regression analysis failed to detect a relationship between age and amplitude for wildtype ChIs [R2 = 0.04886, F_(1, 29)_ = 1.387, *p* = 0.2492, *n* = 29, 9 mice], eIPSC amplitude was clearly related to age in zQ175^+/−^ ChIs [R2 = 0.2076, F_(1, 35)_ = 8.648, *p* < 0.01, *n* = 35, 13 mice].

### Enhanced GABAergic Signaling Had a Presynaptic Locus

**Figure 3:**
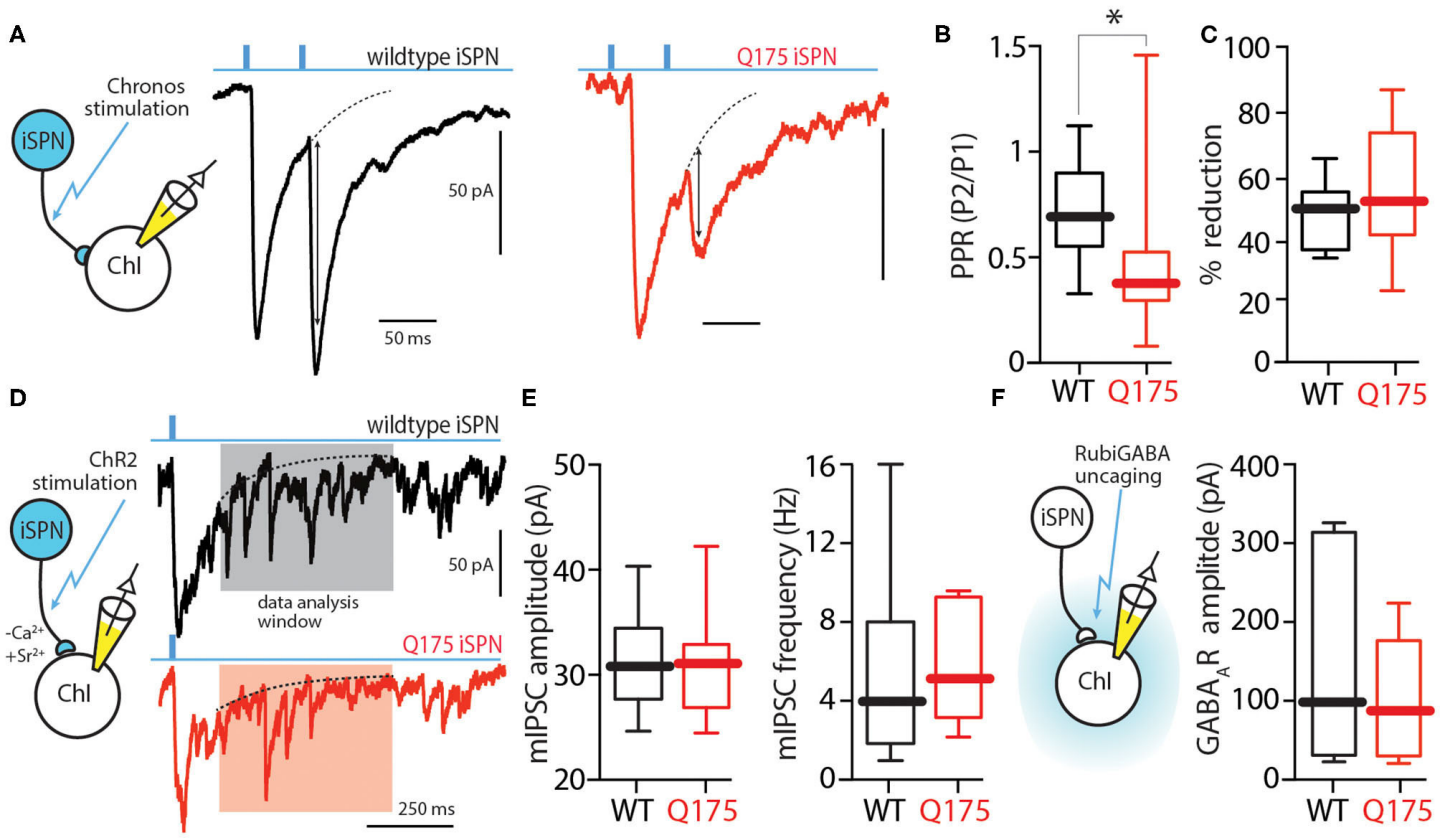
Presynaptic locus of change resulting in enhanced iSPN-ChI GABA signaling. **(A)** Sample traces showing two 0.5 ms long 470 nm light pulses 50 ms apart (20 Hz) and the resulting inward currents in WT (black) and zQ175^+/−^ (red) ChIs. **(B)** Box plots showing medians of the PPR in ChIs from WT and zQ175^+/−^ mice. PPR is defined as peak amplitude of 2nd eIPSC/peak amplitude of the 1st eIPSC. **(C)** Application of the D2 receptor agonist quinpirole (1 μM) causes a decrease in eIPSC amplitude at the iSPN-ChI synapse in both WT and zQ175^+/−^ mice. **(D)** Strontium-evoked asynchronous GABA release amplitude is unchanged in zQ175^+/−^ mice. Replacement of calcium with strontium results in asynchronous neurotransmitter release. Sample traces of optogenetically evoked events in ChIs taken from WT and zQ175^+/−^ mice. Events smaller than 5x RMS of the noise were eliminated from analysis. **(E)** Box plots showing median amplitude and frequency of evoked asynchronous IPSCs for WT and zQ175^+/−^ mice. **(F)** GABA current elicited at ChIs from RuBiGABA uncaging. Box plots showing medians of GABAA receptor currents following uncaging. *P < 0.05.

One potential explanation for the change in iSPN-evoked responses in zQ175^+/−^ ChIs is that the release probability at collateral synapses was elevated. Release probability is usually measured by delivering two stimuli that are closely spaced in time (e.g., 50 ms) and then measuring the amplitude of evoked responses; the ratio of the second response to the first one (P2/P1 - paired pulse ratio or PPR) yields a convenient measure of release probability (Zucker and Regehr, 2002). An obstacle to this assay using ChR2 is that it desensitizes quickly and only slowly recovers, making the interpretation of the response to repetitive stimulation problematic. To overcome this limitation we used a different opsin, Chronos, that recovers more quickly; cells expressing Chronos are able to follow optical stimuli at up to 40 Hz with high fidelity (Klapoetke et al., 2014). Using an AAV9-hSyn-FLEX-Chronos-GFP construct, Chronos was expressed in iSPNs of wildtype and zQ175^+/−^ mice as described above. In *ex vivo* slices prepared from these mice, ChIs were subjected to whole cell recordings using the same Cs^+^-based internal solution described above. Optically evoked eIPSCs were obtained using a 50 ms inter-stimulus interval and PPRs computed. The median PPR (P2/P1) in wildtype ChIs was 0.7 (*n* = 14 cells, 8 mice), whereas in zQ175^+/−^ ChIs it was 0.4 (*n* = 11 cells, 6 mice; Mann Whitney U = 31, *P* < 0.05 two tailed; Figures 3A,B). This decrease in PPR clearly suggests that the release probability at iSPN collateral synapses on ChIs was increased in zQ175^+/−^ mice – providing an explanation for the enhanced amplitude of evoked responses.

One potential explanation for the elevation in release probability at zQ175^+/−^ iSPN collateral synapses is that D2 dopamine receptor (D2R) signaling at iSPN terminals is diminished (Ariano et al., 2002; Pavese et al., 2003). However, bath application of the D2R agonist quinpirole (1 μM) decreased the amplitude of iSPN-evoked IPSCs in both wildtype and zQ175^+/−^ ChIs to a similar extent [wildtype median reduction = 50%, zQ175^+/−^ median reduction = 49%; Mann Whitney U = 23, *n*(wildtype) = 6 cells, 3 mice, n(zQ175^+/−^) = 10 cells, 5 mice; *P* = 0.4923 two-tailed; Figure 3C]. This result argues that differences in ambient D2R signaling were not responsible for the enhancement of iSPN collateral signaling to ChIs. Additionally, cannabinoid receptor type 1 (CB1R) activation with WIN-55,212-2 (1 μM) decreased the evoked IPSC amplitude in both wildtype and zQ175^+/−^ mice (data not shown).

To assess whether there was a change in the postsynaptic complement of GABA_A_Rs at collateral synapses, Ca^2+^ (2 mM) in the external bathing solution was replaced with Sr^2+^ (3.2 mM) to induce asynchronous transmitter release after optical stimulation (Behrends and ten Bruggencate, 1998; Rumpel and Behrends, 1999). In the presence of Sr^2+^, optical stimulation of iSPN collaterals evoked both a synchronous IPSC and later asynchronous miniature IPSCs (mIPSCs) in ChIs (Figures 3D,E). The amplitude of these mIPSCs was measured 500 ms after the optical stimulus, when the synchronous response had decayed to 20% of the peak amplitude (Xu-Friedman and Regehr, 2000). The amplitude of mIPSCs evoked in wildtype and zQ175^+/−^ ChIs was not significantly different [wildtype median = 31 pA, zQ175^+/−^ median = 31 pA; Mann Whitney U = 34, *n*(wildtype) = 10 cells, 4 mice; *n*(zQ175^+/−^) = 7 cells, 3 mice; *P* = 0.9252 two-tailed; Figure 3E]. Moreover, the groups did not significantly differ in the frequency of Sr^2+^ mIPSCs (wildtype median = 4.0 Hz, zQ175^+/−^ median = 5.3 Hz; Mann Whitney U = 27; *P* = 0.4654).

These results suggest that the augmentation of iSPN-evoked IPSCs in zQ175^+/−^ ChIs was not a consequence of enhanced postsynaptic GABA_A_R expression. To provide an independent test of this conclusion, Rubi-GABA (10 μM) was optically uncaged in the extracellular space surrounding ChIs using a blue light pulse (20 ms) while recording postsynaptic currents in ChIs. As with the asynchronous mIPSCs, there was not a significant difference in the uncaging evoked responses in wildtype and zQ175^+/−^ ChIs (wildtype median = 101 pA, *n* = 6 cells, 4 mice; zQ175^+/−^ median = 89 pA, *n* = 7 cells, 4 mice; Mann Whitney U = 19, *p* > 0.05 two tailed; Figure 3F).

### iSPN Activation Suppressed ChI Spiking in HD Mice

**Figure 4:**
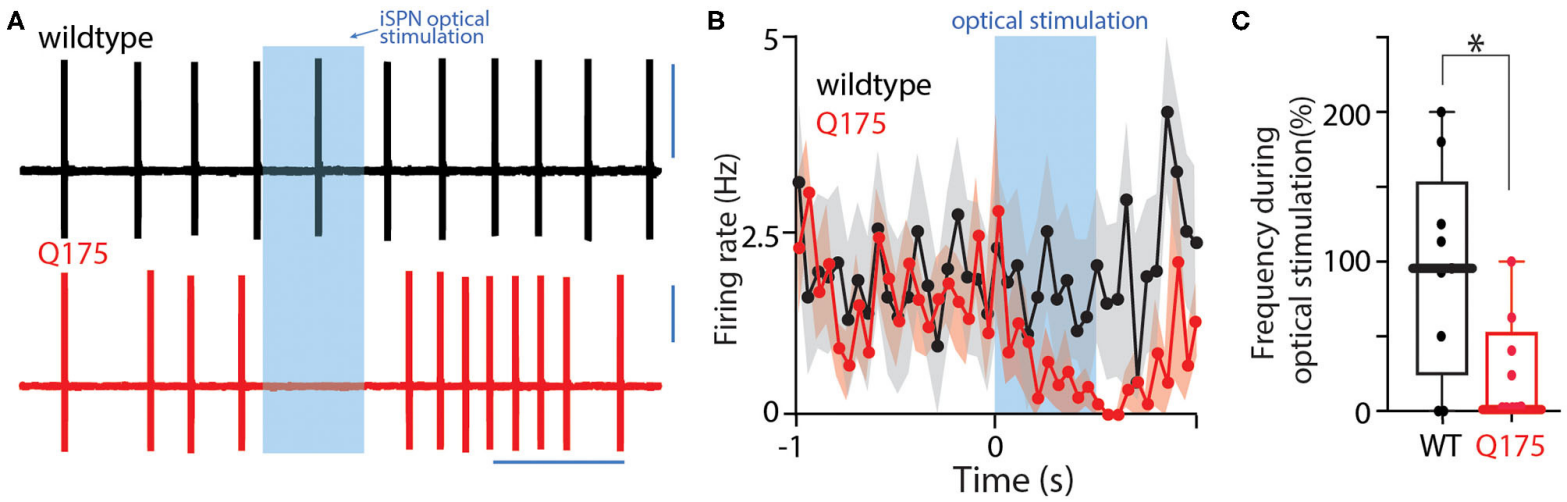
Optogenetic activation of iSPNs inhibits spontaneous ChI firing. **(A)** Sample current clamp recordings from spontaneously firing ChIs in a cell attached configuration. Strong activation of iSPNs induce a pause in activity. **(B)** Peristimulus time histogram of firing rate before, during, and after a high frequency stimulus. Error bars indicate standard error of the mean. **(C)** Boxplot showing that during optogenetic 10-pulse train activation of iSPNs, ChIs from zQ175^+/−^ mice decrease their spontaneous firing rate. *P < 0.05.

Striatal ChIs are autonomous pacemakers that spike at a fairly broad range of frequencies (0.1–10 Hz) (Bennett and Wilson, 1999). Normally, GABAergic synaptic inputs to ChIs are relatively inefficient in pausing this activity (Bennett and Wilson, 1998). To determine if the enhancement of iSPN collateral input to zQ175^+/−^ ChIs changed this situation, ChIs were recorded from in cell-attached mode in *ex vivo* brain slices during optical stimulation of iSPN collaterals. Cell-attached recordings were used to preserve the normal intracellular milieu governing the relationship between GABA_A_R currents and membrane potential. In wildtype and zQ175^+/−^ ChIs, the basal pacemaking rate was very similar (wildtype median = 1.6 Hz, *n* = 9 cells, 4 mice; zQ175^+/−^ median = 1.5 Hz, *n* = 9 cells, 6 mice). As expected from earlier work, optical stimulation of iSPN collaterals was ineffective in altering the rate of WT ChI spiking, even with a 10 pulse burst of stimuli (*n* = 10 cells, 4 mice, Figures 4A,B). However, in zQ175^+/−^ ChIs, the same optical stimulation of iSPNs proved to be very effective in reducing spike rate. The spike rate during iSPN stimulation decreased in all of the zQ175^+/−^ ChIs examined (Figure 4C), falling to zero in four of them (*n* = 9 cells, 6 mice; *P* < 0.01, Mann-Whitney U = 17). Thus, in zQ175^+/−^ mice, iSPNs are able to potently suppress the ongoing, spontaneous spiking of ChIs, in contrast to the situation in wildtype mice.

### NPY Interneuron Input to ChIs Also Was Enhanced in HD Mice

**Figure 5:**
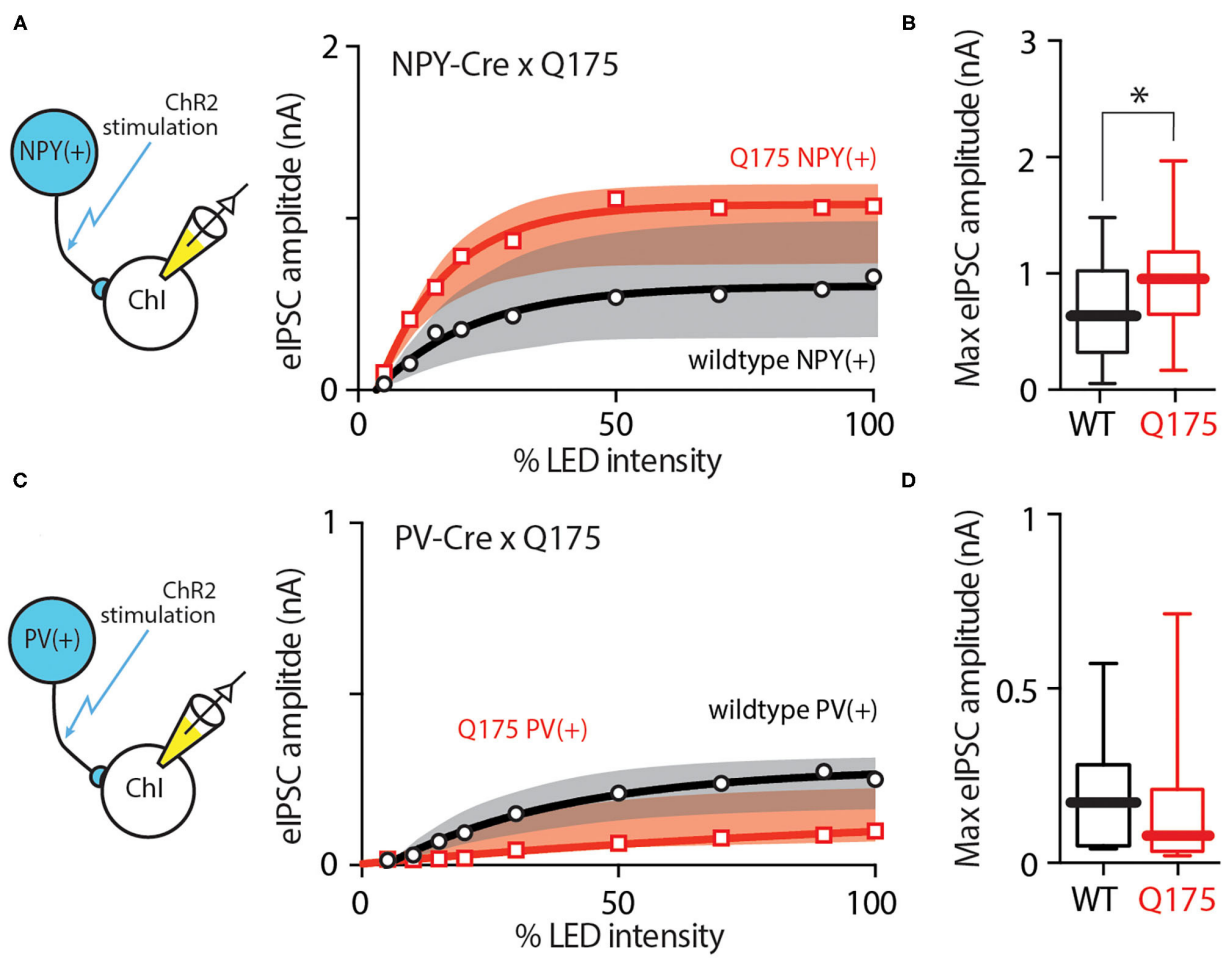
NPY(+) but not PV(+) GABAergic signaling to ChIs was enhanced in zQ175^+/−^ mice. **(A)** Input - output relationship showing enhanced GABA signaling from NPY-expressing cells in ChIs at progressively higher LED intensities. Data from zQ175^+/−^ mice (red) are plotted next to WT (black) mice. **(B)** Box and whiskers plot showing increased eIPSC amplitude at maximum stimulation intensity. **(C)** Input - output relationship and maximum eIPSC amplitude box and whiskers plot showing no difference between WT and zQ175^+/−^ mice at the PV(+) - ChI GABAergic synapse. **(D)** Box and whiskers plot showing no significant difference in eIPSC amplitude at maximum optical stimulation intensity. *P < 0.05.

In addition to the synaptic input from iSPNs, ChIs also receive GABAergic inhibition from other striatal cell populations. One of these is a group of interneurons that express neuropeptide Y (NPY). These interneurons can be divided into two different classes based on morphology and electrophysiological properties: the persistent, low-threshold spiking interneuron (PLTSi) and neurogliaform interneuron (NGFi) (Ibanez-Sandoval et al., 2011). In R6/2 mice, the strength of PLTSi input to ChIs is increased (Holley et al., 2015). To determine if this was also true in the zQ175^+/−^ model, an AAV vector containing a Cre-dependent channelrhodopsin2 expression construct was injected into dorsolateral striatum of NPY-Cre mice crossed into the zQ175^+/−^ line and, after allowing viral expression, optogenetic experiments were performed in *ex vivo* brain slices as described above. In agreement with the earlier work, the eIPSCs in zQ175^+/−^ ChIs were larger than those in wildtype ChIs across a range of stimulus amplitudes (Figure 5A) (wildtype = 14 cells, 7 mice, zQ175^+/−^ = 11 cells, 4 mice). The median eIPSC amplitude evoked by maximal optical intensity stimulation in wildtype ChIs was 660 pA (*n* = 22 cells, 8 mice), whereas the median eIPSC amplitude in zQ175^+/−^ ChIs was 956 pA (*n* = 18 cells, 6 mice; Mann Whitney U = 123, *P* < 0.05 two-tailed; Figure 5B).

To determine if this change extended to other populations of interneurons, parvalbumin (PV)-expressing striatal interneurons were examined. To this end, an AAV vector containing a Cre-dependent channelrhodopsin2 expression construct was injected into dorsolateral striatum of PV-Cre mice crossed into the zQ175^+/−^ line and, after allowing viral expression, optogenetic experiments were performed in *ex vivo* brain slices. In contrast to NPY connections and in agreement with previous work in the R6/2 model (Holley et al., 2015), optical stimulation of PV axons yielded very similar responses in zQ175^+/−^ ChIs and wildtype ChIs (Figure 5C). The median eIPSC amplitude at maximum stimulation intensity in wildtype ChIs was 173 pA (*n* = 16 cells, 4 mice), while it was 76 pA in zQ175^+/−^ ChIs (*n* = 11 cells, 3 mice; Mann-Whitney U = 62, *P* > 0.05 two-tailed; Figure 5D).

## Discussion

A deficit in striatal cholinergic signaling has long been thought to be a contributing factor in the emergence of hyperkinetic HD motor symptoms (Aquilonius and Sjöström, 1971; Vetter et al., 2003; Picconi et al., 2006). Increased GABAergic inhibition of ChIs is one potential driver of this phenotype (Holley et al., 2015). Indeed, in symptomatic zQ175^+/−^ mice, GABAergic iSPN collateral inhibition of ChIs was dramatically enhanced. In contrast to the situation in age-matched, wildtype mice, optogenetic activation of zQ175^+/−^ iSPNs was able to pause on-going, pacemaking activity in ChIs. The enhancement was attributable to an elevation in GABA release probability and not to a change in postsynaptic responsiveness. Importantly, the adaptation was not evident in dSPN collaterals. In addition, the elevation in iSPN collateral strength paralleled the emergence of motor deficits in the zQ175^+/−^ mouse. The elevation in iSPN inhibition of ChIs could make a significant contribution to reducing striatal ACh release in HD models. Moreover, as cholinergic signaling preferentially enhances the responsiveness of iSPNs to excitatory cortical synaptic input (Tanimura et al., 2016), this network adaptation could prevent ChIs from providing temporally appropriate feedback to iSPNs and, in so doing, cut short the indirect pathway activity necessary to suppress unwanted movements, a hallmark of HD (Albin et al., 1990).

### Functional Implications of Altered iSPN Inhibition of ChIs in zQ175^+/–^ Mice

The functional significance of enhanced iSPN inhibition of ChIs in zQ175^+/−^ mice remains to be explored, but there are several factors to consider. One is that under normal circumstances, the impact of the intrastriatal GABAergic circuitry on ChI spiking is very modest (Bennett and Wilson, 1999). In agreement with this assessment, optogenetic stimulation of iSPNs in wildtype tissue had no detectable effect on ChI pacemaking (Figure 4). In sharp contrast, in zQ175^+/−^ slices, iSPN stimulation was able to robustly suppress ongoing spiking. Given that the peak currents evoked by PLTSIs were similar to those evoked by iSPNs (c.f., Figures 2, 5), and that PLTSI excitability is enhanced in zQ175^+/−^ mice (Holley et al., 2019), it stands to reason that both circuits should be able to exert an unusual level of control over the timing of ChI spiking in the HD brain. How these changes might be linked to PLTSi-dependent synaptic plasticity and learning remains to be determined (Rafalovich et al., 2015; Holly et al., 2019).

*But does this happen in vivo*? There is growing evidence that in the early stages of HD pathology the ability of iSPNs to respond to cortical signaling declines (Plotkin and Surmeier, 2015). In part, this may reflect cortical dysfunction (Ross et al., 2014), but it also is a sign of declining postsynaptic responsiveness as iSPN dendritic K^+^ channels become dysregulated and potentiation at glutamatergic synapses is lost (Carrillo-Reid et al., 2019). As iSPNs and the indirect pathway of the basal ganglia help suppress unwanted movement (Bariselli et al., 2019), this deficit in responsiveness and the imbalance with dSPNs provides a ready explanation for the early hyperkinetic phase of the HD phenotype (Albin et al., 1990). In this situation, the up-regulation in GABA release at ChI synapses could reflect a homeostatic adaptation to compensate for declining iSPN activity. Indeed, a similar elevation in synaptic strength occurs at striatopallidal synapses on globus pallidus externa (GPe) neurons (Perez-Rosello et al., 2019). One potential explanation for this change could be a structural remodeling of synaptic connections, as has been described for other striatal interneurons in other movement disorders (Gittis et al., 2011; Yalcin-Cakmakli et al., 2018). In both cases, the change in synaptic properties parallels the emergence of motor deficits in the zQ175^+/−^ model, consistent with the idea that it is a compensation rather than a cause.

However, previous studies showing enhanced GABAergic input to ChIs in HD models have suggested that this synaptic change contributes to HD motor dysfunction (Holley et al., 2015; Du et al., 2017). As ChI activity and cholinergic signaling in the striatum have consistently been linked to suppression of unwanted movement and preferential enhancement of iSPN excitability (Do et al., 2012; Lv et al., 2017), an aberrant suppression of ChI activity could be an important factor in the early hyperkinetic phase of HD. In line with this hypothesis, evoked ACh release is attenuated in R6/2 mice (Farrar et al., 2011) and in zQ175 models (unpublished observations). That said, it is unclear whether this shift depends upon increased GABAergic signaling. One of the hallmarks of the HD striatum is loss of proteins associated with acetylcholine (ACh) release by ChIs; expression of both the synthetic enzyme for ACh synthesis—choline acetyltransferase—and the vesicular transporter for ACh (vAChT) are down-regulated in HD striata (Bird and Iversen, 1974; Smith et al., 2006). Thus, there are other, possibly cell autonomous, mechanisms that could explain the deficit in cholinergic signaling seen in HD models.

One way to resolve whether changes in iSPN connectivity with ChIs or ACh release by ChIs are cell autonomous effects mediated by mutant huntingtin (mHtt) or network-driven adaptations to pathology elsewhere is to employ new genetic approaches. For example, adenoassociated virus (AAV) vectors have recently been used to deliver zinc finger proteins (ZFPs) that specifically target mHtt. Pan-cellular (Carrillo-Reid et al., 2019) or cell-specific expression of mHtt-targeted ZFPs (Zeitler et al., 2019) effectively reversed some features of the HD phenotype in the zQ175^+/−^ model. The availability of cre-recombinase mouse lines that would allow for iSPN- or ChI-specific mHtt-ZFP expression puts these questions within experimental reach.

## Conclusions

Using electrophysiological and optogenetic approaches in *ex vivo* brain slices from wildtype and zQ175^+/−^ mice, a several fold enhancement of iSPN (but not dSPN) GABAergic inhibition of ChIs was discovered. A similar enhancement was found in the projections of PLTSIs, but not FSIs. This cell-specific alteration in synaptic transmission appeared in parallel with the emergence of motor symptoms in the zQ175^+/−^ model. The adaptation had a presynaptic locus, as it was accompanied by a reduction in paired-pulse ratio, but not a change in mEPSC amplitude. These results suggest that compensatory changes in iSPN collateral signaling reduce ChI activity, potentially contributing to the network dysfunction underlying the hyperkinetic phase of HD.

## Data Availability Statement

The raw data supporting the conclusions of this article will be made available by the authors, without undue reservation.

## Ethics Statement

The animal study was reviewed and approved by Northwestern University Animal Care and Use Committee.

## Author Contributions

SL conducted experiments, wrote the manuscript, and prepared figures. DS designed and directed the experiments, wrote the manuscript, and prepared figures. Both authors contributed to the article and approved the submitted version.

## Conflict of Interest

The authors declare that the research was conducted in the absence of any commercial or financial relationships that could be construed as a potential conflict of interest.
