# Supplementary figures and images for: Enhanced GABAergic Inhibition of Cholinergic Interneurons in the zQ175+/− Mouse Model of Huntington's Disease
Source: Front Syst Neurosci. 2021 Jan 20;14:626412. doi: 10.3389/fnsys.2020.626412 (PMC7854471; doi:10.3389/fnsys.2020.626412)

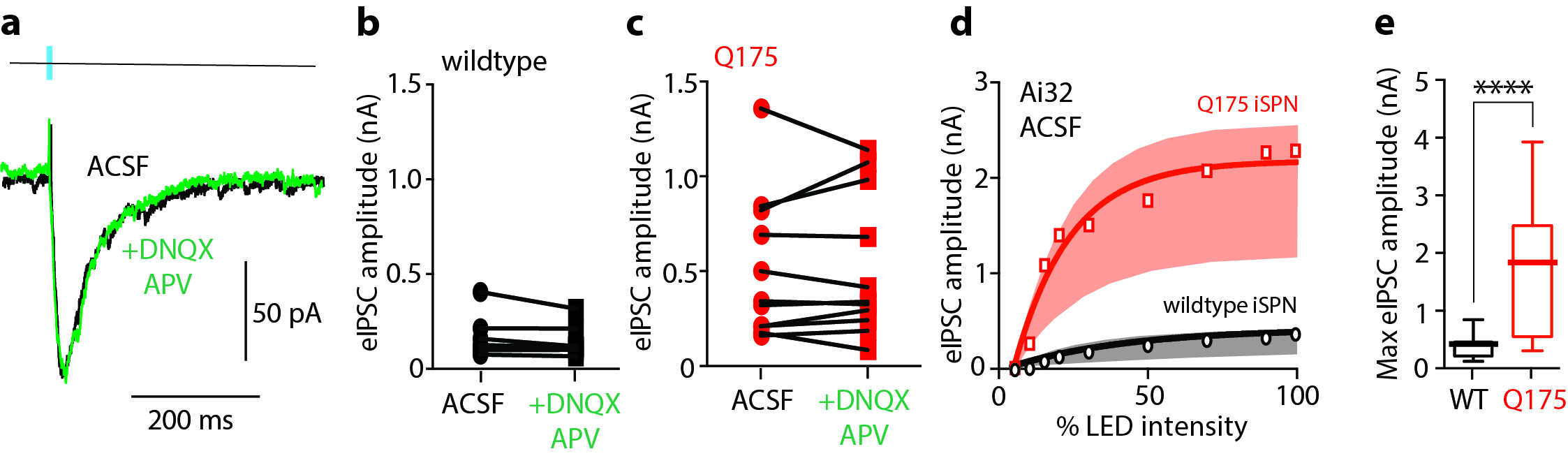

Supplement: Supplementary file 1 [file Image_1.JPEG]
